# Prince of Wales's Hospital Fund for London

**Published:** 1898-12-03

**Authors:** 


### PRINCE OF WALES'S HOSPITAL FUND FOR LONDON.

The following statement has been issued under the signatures of Sir Savile Crossley and Mr. J. G. Craggs, honorary secretaries:—

"It is the earnest desire of His Royal Highness the President, and the Council of the Prince of Wales's Hospital Fund, to secure the widespread co-operation of the community of London in the support of the London hospitals. The merits of these institutions are so well-known and appreciated that we need not describe them; their benefits must have been felt, either directly or indirectly, by every reader of this letter. We feel, however, that when asking you to contribute to the Fund we ought to send you a brief statement of what the Fund did during the first year of its existence, and of what the Prince of Wales wishes to do in the future; which statement, we feel sure, will satisfy you that your loyal and generous support may rightly be given to this Fund.

"Should you have received a previous application this year, we trust that you will forgive us for troubling you a second time, as you will readily understand that, where appeals are necessarily sent out on a large scale, it is very difficult to avoid duplication.

"We wish to draw your attention to two facts. The first is that the Fund has come to stay. The amount of good it does will depend upon the amount of money placed at the disposal of His Royal Highness and the Council, but the success of the Fund has already been so great, and the administration has been so economical, that its permanency may be said to be guaranteed. The second fact is that, so far from the Fund having exercised an injurious effect on the contributions to the various London hospitals, the exact reverse is the case. The ordinary income of the 93 Metropolitan hospitals rose, in 1897, by £35,000, the legacies showed an increase of £62,000, while in the donations for special purposes there was a rise of £55,000.

"Our first annual report and accounts (February 5th to December 31st, 1897) show the total collections to have been £227,551 12s. 5d.

"Amount Distributed to Hospitals.

| | | | |
|---|---|---|---|
| "Annual grants ... ... ... | £22,050 | 0 | 0 |
| "Special donations in honour of Her Majesty's Diamond Jubilee ... | 34,776 | 5 | 0 |
| | "£56,826 | 5 | 0 |

"The annual grants have been allocated in such a way as to provide for the re-opening of about 180 beds hitherto closed in some of the important hospitals for want of means. This, in itself, is equivalent to the establishment of a large new hospital, and has been effected without the great capital outlay which the establishment of a new hospital would entail. Other institutions have also been enabled to increase their efficiency.

"The known annual subscriptions, included in the above collections, are slightly over £21,000 per annum. With the income from investments, about £25,000 per annum may be said to be secured to the Fund, but, as it is desired to secure about £100,000 per annum, by annual subscriptions and yearly collections, further generous support is urgently needed.

"During the present year every effort has been made to extend the operations of the Fund. Special investigations have been made respecting the wants and merits of the Metropolitan hospitals, and a committee has visited and reported fully on the condition of the institutions applying for a grant from the Fund.

"Unnecessary expenditure on administration has been carefully avoided, and last year all costs were covered by £1 12s. 6d. per cent. on the amounts received.

"The printed report, containing full list of subscribers and donors, can be had on application, price 6d.

"If every inhabitant of the metropolis would give even one shilling to the Fund annually, the most sanguine anticipations of the Prince of Wales would be attained."

